# Optimal diagnosis and management of non-alcoholic fatty liver disease

**DOI:** 10.2478/abm-2022-0001

**Published:** 2022-02-28

**Authors:** Editorial Office of Asian Biomedicine

**Affiliations:** Faculty of Medicine, Chulalongkorn University, Bangkok 10330, Thailand

Non-alcoholic fatty liver disease (NAFLD) refers to a spectrum of liver disorders, defined by the presence of steatosis or fat in the liver cells in >5% of hepatocytes of patients after little or no alcohol consumption [1]. Up to 25% of the global population is estimated to have NAFLD, which can be classified as non-alcoholic fatty liver that is benign, or when fat accumulates in hepatocytes, is called liver steatosis or non-alcoholic steatohepatitis (NASH). NASH may progress to cirrhosis and hepatocellular carcinoma [2].

NAFLD can occur in people who take in more calories than the body needs, particularly those with sedentary lifestyle. Type 2 diabetes, dyslipidemia, insulin resistance, and high blood pressure are associated with an increased risk of NAFLD. Therefore, NAFLD has also been regarded as a hepatic manifestation of metabolic syndrome [3], which in turn is associated with an increased risk of developing numerous other health problems and diseases [4].

Liver biopsy is the criterion standard for diagnosis of NAFLD [5]. However, the grading of the severity of NAFLD can be based on the grade of fatty liver from ultrasonography, which can produce increased echogenicity on ultrasound, decreased hepatic attenuation on computed tomography, or an increased fat signal on magnetic resonance imaging [6]. Patients often seek further workup, including laboratory tests and imaging studies, when liver function screening reveals elevated liver aminotransferases [7].

It is important to identify NASH, the more aggressive form of NAFLD. NASH has become a leading reason for liver transplantation in some countries, overtaking cirrhosis from hepatitis and alcohol consumption.

NASH is an important condition that needs early recognition and intervention to slow the progress of the disease, such as through diet and lifestyle modification, and control of obesity, dyslipidemia, hypertension, and other factors associated with metabolic syndrome. Although some have suggested that treatment with vitamin E and pioglita-zone may reduce the progress to steatosis and inflammation [8], vitamin E is not used as a first line treatment for fatty liver disease. It is used only when nondrug treatment fails to improve the fatty liver disease, because vitamin E, in an inappropriate dosage, may also harm the liver. Data concerning the effect of vitamin E on hepatic histology are still lacking. Moreover, the short duration of trials limits conclusions about its safety and efficacy [9]. Moreover, vitamin E treatment has had no effect on fibrosis, which is the strongest indicator of undesirable presence of NASH.

Serum uric acid level is associated with NAFLD independent of other metabolic factors, and this suggests that serum uric acid levels may be used to assess the severity and progression of NAFLD as an alternative in settings where ultrasonography or FibroScan of the liver are not readily available [10]. In addition, serum uric acid is known as an independent risk factor for cardiovascular diseases, including insulin resistance, which is considered as an important risk factor for the development of NAFLD [11]. In NAFLD patients, hyperuricemia is independently associated with the severity of liver damage, which, together with insulin resistance, is a therapeutic target for potential intervention trials [12].

Park et al. [13] have reported in this (February 2022) issue of Asian Biomedicine about the association between serum uric acid levels and fatty liver. They found that serum uric acid level has a stronger association with hepatic steatosis than hepatic fibrosis. In other words, serum uric acid levels may help identify the stage of progression of NAFLD before hepatic fibrosis occurs, which may provide time for effective intervention to slow the progression of NAFLD. The finding is consistent with those of another study that showed serum uric acid levels decreased with the progress of fibrosis in patients with NAFLD [14], suggesting that serum uric acid level is correlated with the quantity of fat in the liver. However, participants of the AASLD/EASL Workshop have not yet included the use of serum uric acid level for assessment of the severity and progression of NAFLD [15].

The present study by Park et al. [13] has highlighted the potential role of serum uric acid level measurement in the management of NAFLD, particularly in areas where ultrasonography or FibroScan of the liver is limited and liver biopsy is deemed too risky. Ideally, the value of serum uric acid level as a substitute for, or in addition to, ultrasonography should be studied further using methodology acceptable to gastrointestinal colleagues, to document the relative value and complementarity of the 2 modes of investigations in the management of NAFLD, which is rapidly becoming an important global burden of disease with implications for the appropriate preparation of health systems in response to the increased global challenge. Any reason to doubt the accuracy of the findings must be carefully addressed by multidisciplinary experts.

## References

[1] Cobbina E, Akhlaghi F (2017). Non-alcoholic fatty liver disease (NAFLD) – pathogenesis, classification, and effect on drug metabolizing enzymes and transporters. Drug Metab Rev.

[2] Huang DQ, El-Serag HB, Loomba R (2021). Global epidemiology of NAFLD-related HCC: trends, predictions, risk factors and prevention. Nat Rev Gastroenterol Hepatol.

[3] Huang TD, Behary J, Zekry A (2020). Non-alcoholic fatty liver disease: a review of epidemiology, risk factors, diagnosis and management. Intern Med J.

[4] Puri P, Sanyal AJ (2012). Nonalcoholic fatty liver disease: definitions, risk factors, and workup. Clin Liver Dis (Hoboken).

[5] Sumida Y, Nakajima A, Itoh Y (2014). Limitations of liver biopsy and non-invasive diagnostic tests for the diagnosis of nonalcoholic fatty liver disease/nonalcoholic steatohepatitis. World J Gastroenterol.

[6] Newsome PN, Sasso M, Deeks JJ, Paredes A, Boursier J, Chan WK (2020). FibroScan-AST (FAST) score for the non-invasive identification of patients with non-alcoholic steatohepatitis with significant activity and fibrosis: a prospective derivation and global validation study. Lancet Gastroenterol Hepatol.

[7] Kalas MA, Chavez L, Leon M, Taweesedt PT, Surani S (2021). Abnormal liver enzymes: a review for clinicians. World J Hepatol.

[8] Sumida Y, Yoneda M (2018). Current and future pharmacological therapies for NAFLD/NASH. J Gastroenterol.

[9] Amanullah I, Khan YH, Anwar I, Gulzar A, Mallhi TH, Raja AA (2019). Effect of vitamin E in non-alcoholic fatty liver disease: a systematic review and meta-analysis of randomised controlled trials. Postgrad Med J.

[10] Sirota JC, McFann K, Targher G, Johnson RJ, Chonchol M, Jalal DI (2013). Elevated serum uric acid levels are associated with non-alcoholic fatty liver disease independently of metabolic syndrome features in the United States: liver ultrasound data from the National Health and Nutrition Examination Survey. Metabolism.

[11] Ryu S, Chang Y, Zhang Y, Kim SG, Cho J, Son HJ (2012). A cohort study of hyperuricemia in middle-aged South Korean men. Am J Epidemiol.

[12] Petta S, Cammà C, Cabibi D, Di Marco V, Craxì A (2011). Hyperuricemia is associated with histological liver damage in patients with non-alcoholic fatty liver disease. Aliment Pharmacol Ther.

[13] Park H, Park K-Y, Kim M, Park H-K, Hwang H-S (2022). Association between serum uric acid level and non-alcoholic fatty liver disease. Asian Biomed (Res Rev News).

[14] Yoneda M, Thomas E, Sumida Y, Imajo K, Hyogo H, Fujii H (2014). Uric acid levels decrease with fibrosis progression in patients with nonalcoholic fatty liver disease. Clin Biochem.

[15] Rinella ME, Tacke F, Sanyal AJ, Anstee QM, Participants of the AASLD/EASL Workshop (2019). Report on the AASLD/EASL Joint Workshop on Clinical Trial Endpoints in NAFLD. Hepatology.

